# Phosphorus Substitution Preference in Ye’elimite: Experiments and Density Functional Theory Simulations

**DOI:** 10.3390/ma14195874

**Published:** 2021-10-07

**Authors:** Jiuye Zhao, Jiazhi Huang, Chunyang Yu, Chunyi Cui, Jun Chang

**Affiliations:** 1 College of Transportation Engineering, Dalian Maritime University, Dalian 116026, China; zhaojiuye@dlmu.edu.cn (J.Z.); hjz@dlmu.edu.cn (J.H.); yuchunyang@dlmu.edu.cn (C.Y.); 2 Faculty of Infrastructure Engineering, Dalian University of Technology, Dalian 116024, China; mlchang@dlut.edu.cn

**Keywords:** density functional theory, ye’elimite, dopant, phase transitions, Rietveld method

## Abstract

Density functional theory (DFT) simulation has been recently introduced to understand the doping behavior of impurities in clinker phases. P-doped ye’elimite, a typical doping clinker phase, tends to form when phosphogypsum is used to manufacture calcium sulfoaluminate cement (CSA) clinkers. However, the substitution mechanism of P has not been uncovered yet. In this study, the influence of different doping amounts of P on the crystalline and electronic structure of ye’elimite was investigated using backscattered scanning electron microscopy–energy X-ray dispersive spectroscopy, X-ray diffraction tests, Rietveld quantitative phase analysis, and DFT simulations. Furthermore, the substitution preference of P in ye’elimite was revealed. Our results showed that increasing the doping amount of P increased the impurity contents in CSA clinkers, transforming the ye’elimite crystal system from the orthorhombic to the cubic system and decreasing the interplanar spacing of ye’elimite. Based on the calculation results of the defect formation energies, additional energies were required for P atoms to substitute Ca/Al atoms compared with those required for P atoms to substitute S atoms in both orthorhombic and cubic systems of ye’elimite. Combined calculation results of the bond length–bond order and partial density of states showed that the doped P atoms preferably substituted S atoms; the second possible substituted atoms were Al atoms, while there was only a slight possibility for substitution of Ca atoms. The substitution of P atoms for S atoms can be verified based on the elemental distribution in P-doped ye’elimite and the increasing residual CaSO_4_ contents. The transition of the crystal system and a decrease in the interplanar spacing for ye’elimite can also prove that the substitution of P atoms for Al atoms occurred substantially.

## 1. Introduction

Ye’elimite, also known as tetracalcium trialuminate sulfate, is the dominant mineral in calcium sulfoaluminate (CSA) cement and exhibits high tailored dimensional expansion and early strength [1,2,3]. Since the 1970s, CSA cement has been widely used as a rapid repairing material and has recently been promoted for use in numerous domains, including soft soil stabilization and three-dimensional printing [4,5,6]. Moreover, CSA cement has been considered a promising alternative binder owing to its lower CO_2_ emissions and energy consumption compared with Portland cement, in which ye’elimite plays an essential role [7,8,9]. The forming conditions of ye’elimite determine the sintering temperatures and prolong time of CSA clinkers, while the main hydration products of CSA cement are produced by reactions between ye’elimite and gypsum [10,11].

The chemical formula of stoichiometric ye’elimite is Ca_4_Al_6_SO_16_ which has been identified as an orthorhombic system with a Pcc2 space group at room temperature using diffraction tests and Rietveld method [12]. However, since ye’elimite is a sodalite with a general composition of M_4_(T_6_O_12_)X, certain types of dopants can enter its lattice during the practical manufacturing process of CSA clinkers, forming solid solutions of ye’elimite [13,14]. The solid solutions of ye’elimite would form easily when solid waste was used as raw materials for manufacturing CSA clinkers [15]. Cuesta et al. investigated the crystalline properties and hydration process of a typical solid solution of ye’elimite with the chemical formula of Ca_3.8_Na_0.2_Al_5.6_Fe_0.2_Si_0.2_SO_16_, which is a cubic system with an I43m space group at room temperature [16,17]. Chang and Zhao et al. investigated the formation kinetics, crystalline transition, and hydration properties of a series of solid solutions of ye’elimite with chemical formulas of Ca_4−x_(Sr/Ba)_x_Al_6_SO_16_ (x represents the doping amount) to use strontic/baric slags as raw materials for synthesizing CSA clinkers [18,19,20]. Bullerjahn et al. found that compared with stoichiometric ye’elimite, the formation of Ca_4_Al_6−x_Fe_x_SO_16_ was accelerated by the fluxing and mineralizing effect of iron ions. However, the thermal stability of Ca_4_Al_6−x_Fe_x_SO_16_ was reduced [13].

On the other hand, it can be noticed that density functional theory (DFT) simulations have been recently introduced to understand the doping behavior of impurities in clinker phases [21,22]. Meanwhile, the structural stability and hydration mechanism of clinker phases can be also revealed through DFT [23,24]. As for CSA clinker phases, substitution preference of impurities, including Cu, Pb, and Ba in ye’elimite, belite, and ferrite was investigated by comparing formation energies [25,26,27]. Generally, the results of DFT simulations can adequately accord with experimental phenomena, which can also explain the mechanism of phase transitions and hydration for clinker phases based on the first principles [21,22,23,24,25,26,27].

Alternatively, some studies have sought solutions for recycle of phosphogypsum which is a type of solid waste discharged at more than 150 million tons per year globally [28,29]. Since the composition of phosphogypsum is similar to that of natural gypsum, its use in synthesizing CSA clinkers has been considered a viable option [30]. Compared with natural gypsum, phosphogypsum contains slight amounts (around 1.0wt.%) of phosphorous impurities that enter ye’elimite and affect the synthesized CSA clinkers [31,32]. Previous studies have focused on the composition, formation process, hydration characteristics, and performance of CSA clinkers prepared using phosphogypsum [29,31,33]. However, the substitution preference of P in ye’elimite and the doping mechanism have not been investigated. Additionally, it should be noted that the doping behaviors of impurities in ye’elimite with different crystal systems have not been compared yet, which might lead to neglecting potential phase transitions caused by the doping effect.

In this study, the influence of different doping amounts of P on the crystalline and electronic structure of ye’elimite was investigated and the doping mechanism was revealed by combining experiments with DFT simulations. Backscattered scanning electron microscopy–energy X-ray dispersive spectroscopy (BSEM–EDS) was used to determine the elemental distribution in P-doped ye’elimite, while X-ray diffraction tests and Rietveld method were performed for crystalline analysis. The defect formation energy (*E*_f_) of P-doped ye’elimite with different configurations was calculated and compared using DFT simulations. Moreover, intrinsic factors influencing the doping preference were determined using the partial density of states (PDOSs) and bond length–bond order (BL–BO).

## 2. Experiments and DFT Simulations

### 2.1. Experiments

Analytical reagents, namely, CaCO_3_, CaSO_4_, Fe_2_O_3_, SiO_2_, Al_2_O_3_, Na_2_CO_3_, and Ca_3_(PO_4_)_2_, were used as raw materials for synthesizing different types of ye’elimite, including stoichiometric ye’elimite (Ca_4_Al_6_SO_16_, orthorhombic system), a typical solid solution ye’elimite (Ca_3.8_Na_0.2_Al_5.6_Fe0.2Si_0.2_SO_16_, cubic system), and P-doped ye’elimite. A planetary ball mill was used to homogenize a mixture of raw materials before it was placed in a high-temperature furnace in the form of compressed cylindroid samples (Φ5.0 cm × H1.0 cm) for sintering. Table 1 lists the nomenclatures, molar ratios of raw materials, and sintering conditions of different types of ye’elimite.

P-doped ye’elimite clinkers were polished and sprayed with gold for BSEM–EDS analysis. A scanning electron microscope (FEI Quanta 450, FEI, Hillsboro, OR, USA) was used with an acceleration voltage of 20 kV. All synthesized clinkers were ground to pass via 80-μm sieves for XRD tests using an X-ray diffractometer (Bruker D8 advance Davinci design) with Cu Kα_1,2_ radiation (λ_1_ = 1.5406 Å and λ_2_ = 1.5444 Å). Diffraction data were collected at 5°–80° (2θ) in the step-scan mode with an operating power of 1600 W (operating voltage was 40 kV, and current was 40 mA). The step size was 0.02°, and the step duration was set to 0.5 s. To determine the phases in tested clinkers using the crystallography open database (COD) 2013, the Bruker evaluation software was used to analyze the obtained XRD patterns. Thereafter, the Rietveld method was conducted using TOPAS4.2, where the parameters were set according to our previous studies [18,19,20]. Table 2 presents information on all the phases of Rietveld method.

### 2.2. DFT Simulations

**Figure A1:**
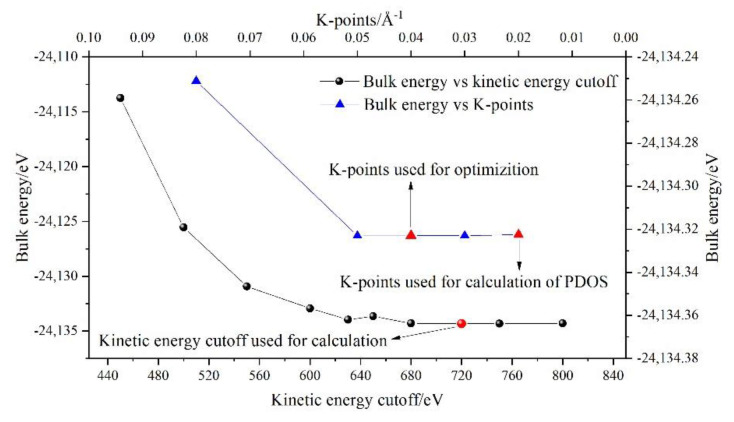
Results of kinetic energy cutoff test and K-points (SS). Structure of SS was selected to conduct the kinetic energy cutoff test and K-points test, as the structure contains all pseudopotentials.

The Cambridge sequential total energy package (CASTEP) [41] was used to optimize the geometry of the model and calculate the total energy (*E*_t_), BL–BO distributions and PDOSs of P-doped ye’elimite based on DFT simulations. The generalized functional gradient approximation was combined with the Perdew–Burke–Ernzerhof (PBE) method to calculate the electron exchange correlation [42]. The projection-augmented wave was used for all simulations, and the kinetic energy cutoff of the plane wave base was set to 720 eV [43] (the results of the kinetic energy cutoff test and K-points can be seen in Figure A1). Considering the calculation accuracy, the convergence energy, force, stress and displacement were determined using 10^−6^ eV/atom, 0.01 eV/Å, 0.02 GPa and 10^−4^ Å, respectively. The lattice relaxation and PDOS calculations were performed on k-point grids with densities of 0.04 and 0.02 (1/Å), respectively. The detailed grids of the two ye’elimite phases are presented in Table A1.

**Figure 1:**
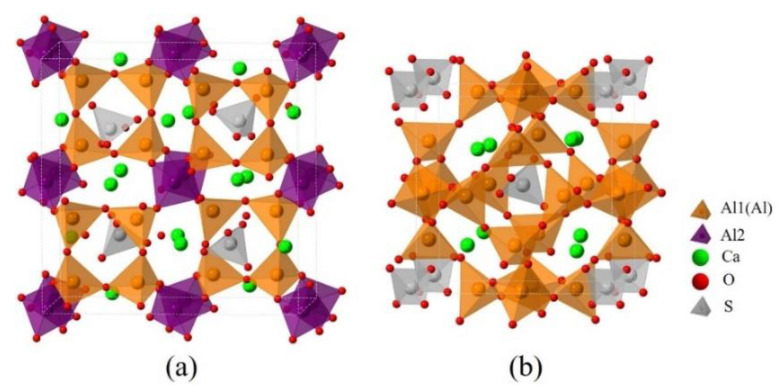
Crystalline structures of ye’elimite: (**a**) orthorhombic (1 × 1 × 1) and (**b**) cubic (1 × 1 × 1) systems.

Since dopants can cause transformation of the crystal system of ye’elimite, both orthorhombic and cubic systems were considered and investigated for comparison in this study. The orthorhombic system of ye’elimite exhibits the Pcc2 space group and contains 64 O, 4 S, 24 Al (according to the symmetry, 12 Al in special positions and labeled Al1, while the remaining 12 Al in general positions and labeled Al2 [44]), and 16 Ca atoms in the unit lattice [12]. In the case of the cubic system of ye’elimite, the space group is I43m and the unit lattice comprises 32 O, 2 S, 12 Al, and 8 Ca atoms [16]. Figure 1 shows the crystalline structures of orthorhombic and cubic systems of ye’elimite, and Table A2 presents the lattice parameters.

Note that the doping mechanism can be classified into two types: interstitial and substitutional. Previous studies have shown that the substitutional type can occur more readily than the interstitial type [45,46,47]. Thus, only P doping substitution has been considered in this study. To ensure an equal number of atoms and the same substitutional ratios are involved in calculations, the cells/supercells with sizes of 1 × 1 × 1 and 2 × 1 × 1 were constructed for the orthorhombic and cubic systems, respectively. It can be noticed that, as all cell/supercell parameters of a, b and c were larger than 9Å (see Table A3), the established cell/supercell totally fulfilled the periodic boundary condition. The crystal structures of pure orthorhombic and cubic ye’elimite used in this paper were obtained through optimizing the crystal structures in Ref. [12] and Ref. [16], respectively. To establish a P-doped model with a cubic system, one P atom was introduced in the crystalline structure of cubic ye’elimite (labeled as P@SS) at the Ca, Al, and S positions (labeled as P@Ca, P@S, and P@Al, respectively). Furthermore, two P atoms were introduced in the crystalline structure of orthorhombic ye’elimite (labeled as P@ST) at the Ca, S, Al1, and Al2 positions (labeled as P@Ca, P@S, P@Al1, and P@Al2, respectively). All established models for P-doped ye’elimite can be seen in Section 3.3 and Section 4.

In this study, the possibility of P doping behavior in ye’elimite was evaluated using *E*_f_, which can be calculated using Equation (1) [48,49,50,51].

(1) $$E_{f}=\frac{1}{n}\left[E_{p}-E_{0}-n\mu_{P}+n\mu_{0}+q(E_{F}+E_{\mathrm{VBM}})\right]$$

where *E*_0_ is the bonding energy of pure ye’elimite, and *E*_P_ is the bonding energy of P-doped ye’elimite (bonding energy refers to the energy required to separate each pseudopotential atom to infinity, and details of the relationship between bonding energy and total energy can be seen in Table A4). *n* is the number of P atoms introduced in ye’elimite, and *q* denotes the net electron number of P-doped ye’elimite (as the raw material used for impurity was Ca_3_(PO_4_)_2_ and the conditions of synthesis were high temperatures, P^5+^ preferred to be the most stable charge state of P in this paper). *E*_F_ represents the Fermi energy relative to *E*_VBM_, where *E*_VBM_ is the energy of the maximum valence band of pure ye’elimite. Furthermore, since the doping rates of P in the orthorhombic and cubic systems of ye’elimite were kept constant in all calculations, normalization was not needed in this study. *μ*_P_ represents the chemical potentials of impurities (P atoms), and *μ*_0_ represents the chemical potentials of substituted atoms (Ca, Al or S atoms). The terms n*μ*_0_-n*μ*_P_ in Equation (1) for different configurations were calculated according to Equations (2)–(4) as below, and details on Gibbs free energies involved in calculations for *μ*_P_ and *μ*_0_ are presented in Table A2.

(2) $$n\mu_{S}-n\mu_{P}=nG_{{\mathrm{SO}}_{3}}-\frac{n}{2}G_{P_{2}O_{5}}-\frac{n}{2}\mu_{O}$$

(3) $$n\mu_{\mathrm{Ca}}-n\mu_{P}=nG_{C\mathrm{aO}}-\frac{n}{2}G_{P_{2}O_{5}}-\frac{3n}{2}\mu_{O}$$

(4) $$n\mu_{\mathrm{Al}}-n\mu_{P}=\frac{n}{2}G_{{\mathrm{Al}}_{2}O_{3}}-\frac{n}{2}G_{P_{2}O_{5}}-n\mu_{O}$$

As for Equations (2)–(4), *μ*_O_ was set as the Gibbs free energy of oxygen in the stable pure bulk single-crystal phase, which can be described by Equation (5).

(5) $$G_{O_{2}}=2\mu_{O}≅2\mu_{O}^{0}$$

## 3. Results

### 3.1. Elemental Distribution in P-Doped Ye’elimite

**Figure 2:**
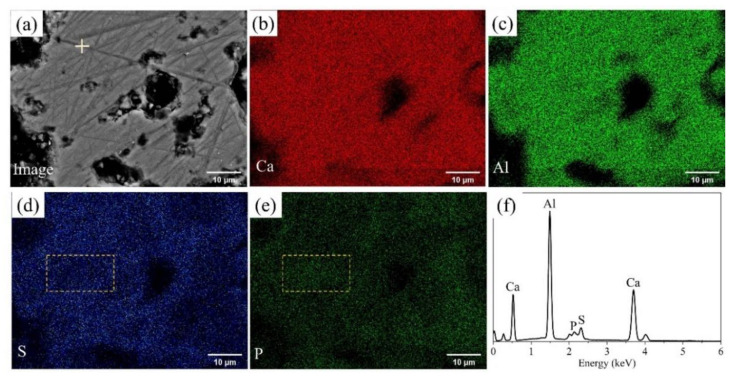
BSEM–EDS results for P0.10: (**a**) Image of polished sample, distributions of (**b**) Ca, (**c**) Al, (**d**) S, and (**e**) P, and (**f**) EDS result for the crossed point in Figure 2a.

BSEM–EDS was performed to determine the elemental distribution in P-doped ye’elimite, and the results of P0.10 are shown in Figure 2. Based on the contrasts observed in Figure 2a and the elemental distribution in Figure 2b–e, P-doped ye’elimite accounted for the vast majority of the observed region. In particular, Ca, Al, S, and P were almost evenly distributed in P-doped ye’elimite, indicating that P entered the lattices of ye’elimite. Further, the densities of S in certain areas were slightly attenuated (such as in the area indicated by the dotted line in Figure 2d), while the densities of P slightly increased in the corresponding areas (such as the area indicated by the dotted line in Figure 2e). This phenomenon suggests that P and S exhibit a potential substitutional relationship in P-doped ye’elimite.

### 3.2. Rietveld Quantitative Phases Analysis of Synthesized Clinkers

**Figure 3:**
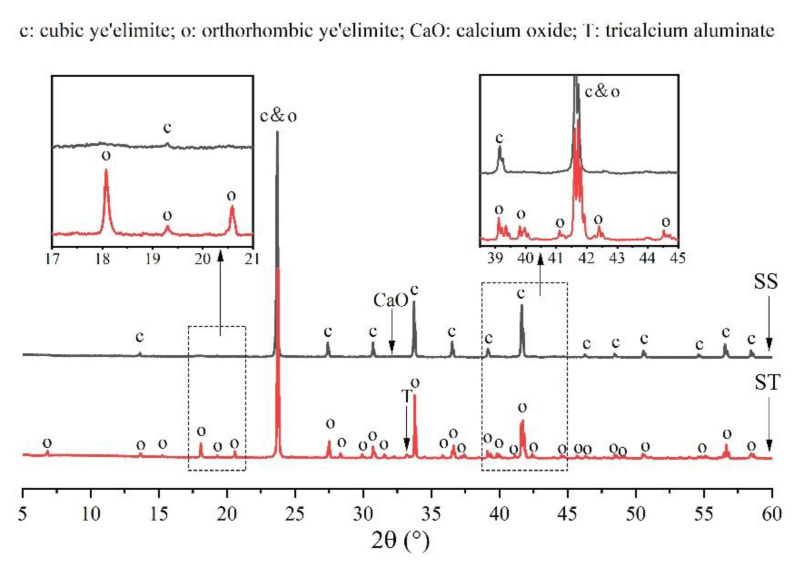
Comparison of XRD patterns of orthorhombic and cubic systems of ye’elimite.

Figure 3 shows a comparison of the XRD patterns of orthorhombic and cubic systems of ye’elimite to determine differences in their crystalline structures. Except for some low-intensity peaks of tricalcium aluminate (Ca_3_Al_2_O_4_) and calcium oxide (CaO), all peaks belonged to the orthorhombic and cubic systems of ye’elimite. Furthermore, the orthorhombic and cubic system patterns were generally similar as the major peaks of both the patterns overlapped at a high intensity (such as the peaks at 23.7°, 33.7°, and 41.6°). However, compared with the cubic system pattern, the orthorhombic system pattern exhibited some additional characteristic peaks (such as those appearing at approximately 18.1°, 20.6°, 35.8°, and 37.3°), which can be observed in the magnified image of Figure 1. Thus, the additional characteristic peaks can be used to distinguish the orthorhombic and cubic systems.

**Figure 4:**
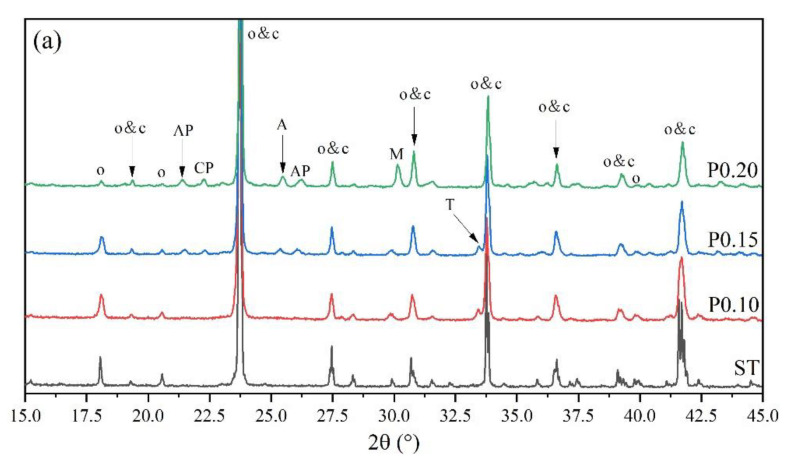

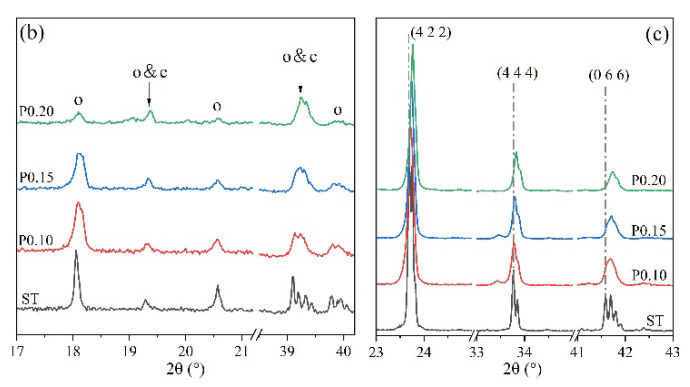
XRD patterns of P-doped ye’elimite, (**a**) identification of phases (o, orthorhombic ye’elimite; c, cubic ye’elimite; A, anhydrite; AP, aluminum phosphate; CP, calcium phosphate; M, monocalcium aluminate; T, tricalcium aluminate), (**b**) comparison of characteristic peaks of orthorhombic ye’elimite, and (**c**) comparison of three major peaks of ye’elimite.

Figure 4 shows the XRD patterns of P-doped ye’elimite in comparison with the orthorhombic system. All diffraction peaks were assigned according to the standard diffraction patterns of ye’elimite (COD#4001772 and COD#4511960), anhydrite (COD#5000040), mayenite (COD#2102955), calcium phosphate (COD#1517238), aluminum phosphate (COD#9006404), monocalcium aluminate (COD#4308075), CaO (COD#7200686), and tricalcium aluminate (COD#9015966).

As shown in Figure 4a, more peaks for impurities, such as Ca_3_(PO_4_)_2_, AlPO_4_, CaAl_2_O_4_, and CaSO_4_, can be clearly detected in samples of P0.15 and P0.20, compared with ST and P0.10 samples. The peak intensities for Ca_3_(PO_4_)_2_, AlPO_4_, and CaSO_4_ in particular gradually increased as the P doping amount increased. Moreover, according to Figure 4b, higher phosphorus doping amounts yielded lower intensities of characteristic peaks for orthorhombic ye’elimite, indicating that ye’elimite gradually transformed from the orthorhombic to the cubic system. Furthermore, based on the comparison of three major peaks of ye’elimite in Figure 4c, the 2θ values for the corresponding peaks increased as the P doping amounts increased. When combined with the equation of Bragg diffraction (Equation (6)), it can be deduced that higher P doping amounts decrease the ye’elimite interplanar spacing.

2*d*sinθ = n*λ* (6)

**Figure 5:**
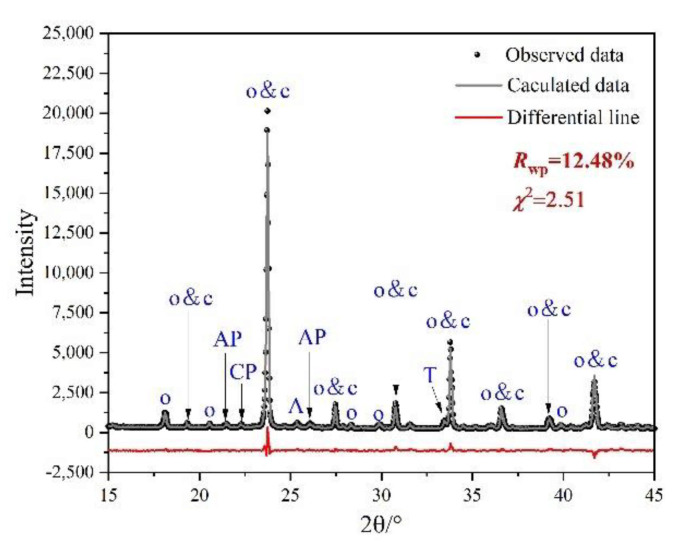
Selected range (15–45°/2θ) of the Rietveld plots for P0.15. Observed data resulted from XRD test, calculated line resulted from Rietveld refinement, and the differential line resulted from the difference between observed data and calculated line.

Rietveld quantitative phase analysis (RQPA) was performed to further determine the compositions of clinkers containing P-doped ye’elimite. Figure 5 shows a selected range (15–45°/2θ) of the Rietveld plots for P0.15, and the results are presented in Table 3. According to the RQPA results, as the P doping amounts increased from 0 mol to 0.20 mol, the total ye’elimite content decreased from 96.4 to 84.6 wt.%, while the impurity contents of calcium aluminate phases and calcium/aluminum phosphate increased from 2.8 and 0 wt.% to 11.6 and 2.7 wt.%, respectively. Note that the CaSO_4_ residues accounted for 0.6 and 1.2 wt.% in P0.15 and P0.20 clinkers, respectively. Additionally, based on the RQPA results, the transformation of the crystal system caused by large P doping amounts can be reflected by the ratio of cubic ye’elimite to orthorhombic ye’elimite.

### 3.3. Defect Formation Energies of P-Doped Ye’elimite

**Figure 6:**
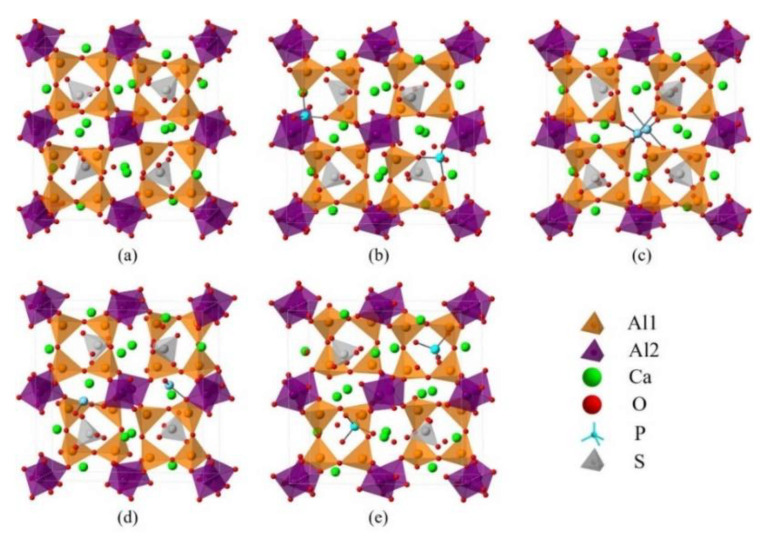
Crystalline structures of orthorhombic ye’elimite (ST) and the doped models (1 × 1 × 1), (**a**) pure, (**b**) P2@Al1, (**c**) P2@Al2, (**d**) P2@Ca, and (**e**) P2@S.

**Figure 7:**
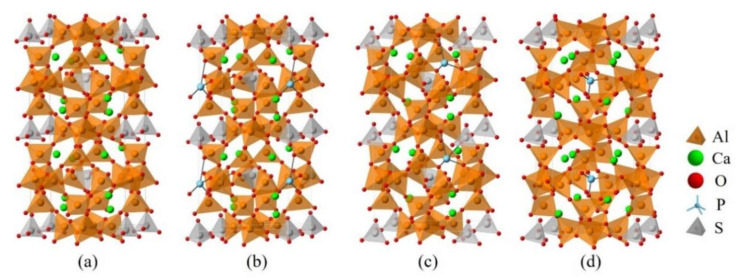
Crystalline structures of cubic ye’elimite and the doped models (2 × 1 × 1), (**a**) pure, (**b**) P@S, (**c**) P@Ca, and (**d**) P@Al.

**Figure 8:**
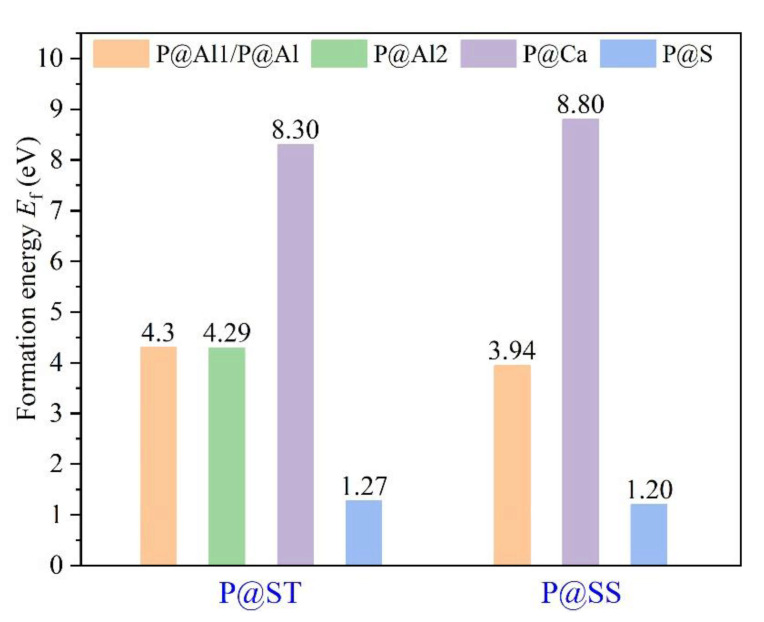
Defect formation energies (*E*_f_, eV) of P-doped ye’elimite with different configurations.

The *E*_f_ of P-doped ye’elimite with orthorhombic and cubic systems was calculated by considering different configurations to determine the substitution preference of P doping in ye’elimite. The doped models used for calculations are shown in Figure 6 and Figure 7, and the lattice parameters of the doped models are presented in Table A3. The calculations *E*_f_ results are shown in Figure 8 (refer to Table A4 for details). The relative stabilities of defects and possibilities of reaction can be reflected by the values of formation energies (*E*_f_), in which lower *E*_f_ represents more stable configuration and the easier reaction [45,47]. Thus, the *E*_f_ values can be used to estimate the possibility and stability of doping behavior. According to the results presented in Figure 8, P atoms most preferentially tended to substitute S atoms since the *E*_f_ values of P@S configurations remained to be minimum (1.27 and 1.20 eV for orthorhombic and cubic systems, respectively). The elemental distribution of P and S obtained using BSEM–EDS can be used to identify the substitution of P atoms for S atoms. Additionally, the increasing CaSO_4_ residue in the clinkers with P-doped ye’elimite can confirm the substitutional relationship between P and S since CaSO_4_ generally cannot be detected in clinkers of pure ye’elimite owing to the inevitable decomposition of CaSO_4_ during the temperature-rise period [52,53].

Alternatively, when P atoms substituted Ca/Al atoms, the *E*_f_ values remained positive and followed the alignment of P@Al (P@SS) < P@Al1 (P@ST) ≈ P@Al2 (P@ST) < P@Ca (P@ST) < P@Ca (P@SS). The alignment indicates that further additional energies were required for P atoms to substitute Ca atoms than for P atoms to substitute Al atoms. However, since P-doped ye’elimite was synthesized at a high temperature (1300 °C in this study), the required additional energies could be easily obtained to overcome the barrier for P atoms to substitute Ca/Al atoms.

### 3.4. Electronic Structural Matching

To further estimate the possibility of different P-doped configurations, the BO–BL distributions in P-doped ye’elimite were calculated according to the Mulliken population rule [54]. Generally, covalent bonds exhibit BO values close to 1, whereas ionic bonds show BO values close to 0. Further, greater overlaps in BO–BL distributions between substituted atoms and dopants indicate a greater possibility for doping behavior to form solid solutions.

**Figure 9:**
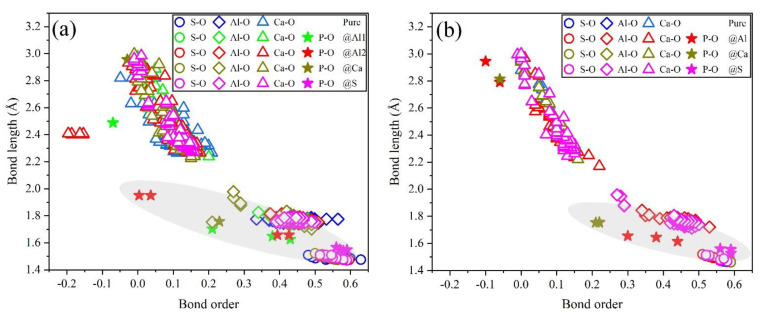
Distributions of BL–BO in pure and P-doped ye’elimite with different configurations: (**a**) P@ST and (**b**) P@SS.

Figure 9 shows the BL–BO distributions in pure and P-doped ye’elimite with different configurations. For both orthorhombic and cubic ye’elimite, S–O bonds were observed with relatively high BO bonds, Ca–O bonds showed BO values of close to 0, and Al–O bonds were located in the regions between S–O and Ca–O bonds. Furthermore, when P atoms were introduced to substitute S atoms in P-doped ye’elimite, P–O bond regions overlapped well with S–O bond regions. In the configurations where P atoms substituted Al atoms, partial P–O bonds approached closely to the regions of Al–O bonds in both orthorhombic and cubic systems (refer to the marked regions in Figure 9a,b). However, when P atoms substituted Ca atoms, most of the P–O bonds remained away from the regions of Ca–O bonds.

**Figure 10:**
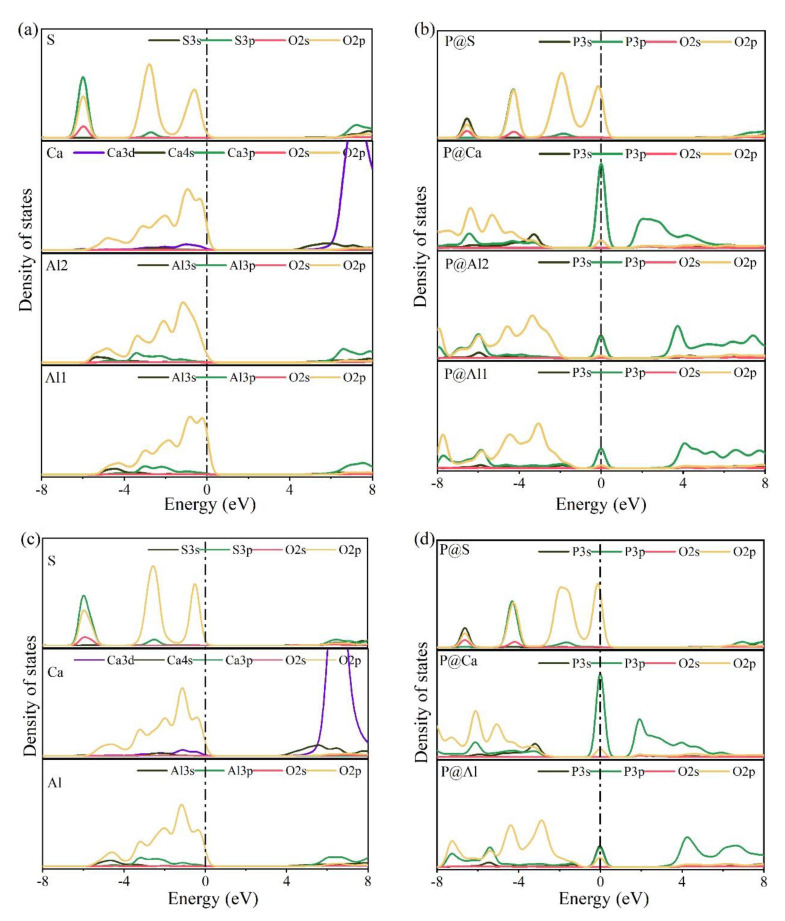
Calculation results of PDOS for different elements in the configurations of pure and P-doped ye’elimite (position of the Fermi energy level (0 eV) is indicated by a dotted line), (**a**) pure orthorhombic system (ST), (**b**) P@ST, (**c**) pure cubic system (SS), and (**d**) P@SS.

The electronic structures of bonds formed between O and substituted/doped atoms can be further investigated using PDOS. Figure 10 shows the PDOS calculation results for various elements in pure and P-doped ye’elimite. According to Figure 10a,c, for pure ye’elimite with both orthorhombic and cubic systems, the O 2p orbitals hybridized with Al 3p and S 3p orbitals to form Al–O and S–O bonds, respectively, while the O 2p orbitals hybridized with Ca 3d orbitals to form Ca–O bonds. To form P–O bonds in P-doped ye’elimite (Figure 10b,d), the O 2p orbitals hybridized with P 3s and P 3p orbitals. Compared with pure ye’elimite, the PDOS of P@S (in both P@ST and P@SS) exhibited the most similar distribution for all P-doped ye’elimite configurations, whereas partial overlapping (between −4 and −2 eV) could be observed for the PDOS of P@Al, including P@Al in P@SS and P@Al1/P@Al2 in P@ST. Additionally, similar electronic contributions between P–O and Ca–O bonds are seldomly observed in both orthorhombic and cubic structures of ye’elimite.

Combining the BL–BO and PDOS calculation results, for P-doped ye’elimite with both orthorhombic and cubic systems, the P atoms preferably substituted S atoms; the second possible substituted atoms were Al, while the substitution for Ca atoms exhibited only a slight possibility.

## 4. Discussion

According to the *E*_f_, BL–BO, and PDOS calculation results, P atoms were more likely to substitute S atoms, which is a spontaneous process, rather than Al/Ca atoms. In addition, the substitution of P atoms for S atoms can be verified using the elemental distribution in P-doped ye’elimite (Figure 2) and the increasing residual CaSO_4_ contents (Table 1).

**Figure 11:**
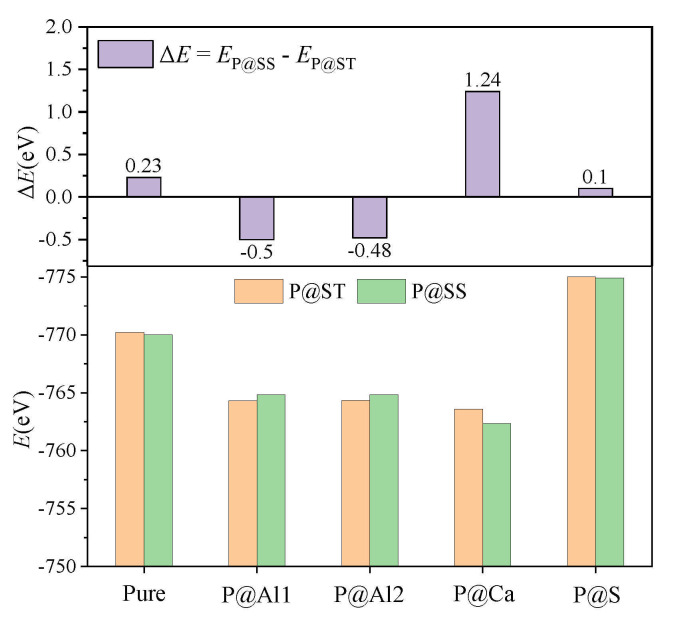
Static energies (*E*, eV) of configurations with P substituting different atoms in P@ST and P@SS.

According to the XRD patterns demonstrated in Figure 4 and the RQPA results presented in Table 1, increasing the P doping amount caused the transformation of the crystal system from the orthorhombic to the cubic system. Previous studies have shown that the freezing of anionic groups caused by dopants prompted the transformation of the crystal system of ye’elimite during cooling [16]. The freezing effect can also be reflected by the *E* values of ye’elimite, where a lower *E* value generally represents a more stable structure that tends to be retained during the crystalline transformation process [12]. To confirm the stability of the P-doped ye’elimite structure, the *E* values for P substituting different atoms in orthorhombic and cubic structures of ye’elimite are compared in Figure 11.

According to the results depicted in Figure 11, for pure ye’elimite, the orthorhombic system demonstrated more stability at the room temperature than the cubic system, which agrees with the experimental results presented in Figure 3. Similarly, for configurations with P atoms substituting Ca/S atoms, the orthorhombic system of P-doped ye’elimite exhibited lower *E* values than the cubic system of P-doped ye’elimite. However, when P atoms were introduced to substitute Al atoms, the *E* value for the cubic system was lower than that for the orthorhombic system. When all configurations with P substituting different atoms in P@ST and P@SS were combined, only the substitution of P atoms for Al atoms would result in the transformation of the crystal system of P-doped ye’elimite.

Additionally, based on the XRD patterns shown in Figure 4, increasing the P doping amount resulted in a gradual decrease in the ye’elimite interplanar spacing, which is generally explained by smaller ionic radii of dopants compared with substituted atoms [14,18,20]. According to the radii of the ions involved in P-doped ye’elimite (Table 4), all the radii of phosphonium ions with different coordination numbers are larger than that of sulphion, implying that the decrease in the ye’elimite interplanar spacing cannot be attributed to the substitution of P atoms for S atoms. Considering the slight possibility of substitution for Ca atoms revealed by BL–BO and PDOS results, the substitution of P atoms for Al atoms was the most likely driving force for the decreasing ye’elimite interplanar spacing.

**Figure 12:**
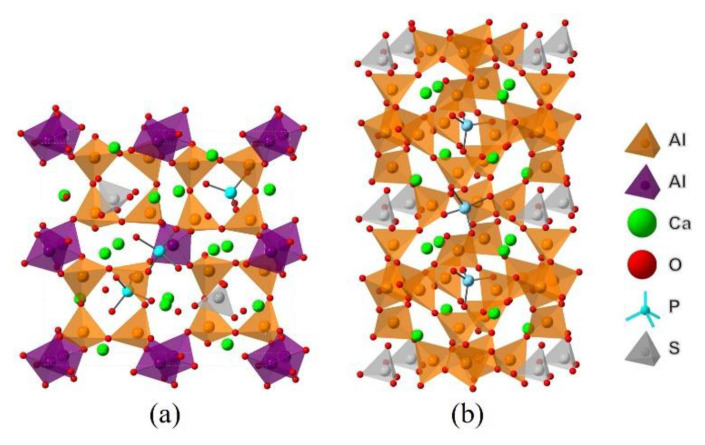
Models of co-substituting P-doped ye’elimite. (**a**) P@S&P@Al(ST), (**b**) P@S&P@Al(SS).

Combining the above analysis with the consideration of charge equilibrium in the lattices, the co-substituting P-doped ye’elimite were proposed to be the most probably models (see Figure 12), in which P atoms substituted two S atoms and one Al atom in cell/supercell of orthorhombic and cubic ye’elimite, respectively (P@S&P@Al(ST) refers to the co-substituting P-doped ye’elimite with orthorhombic system, P@S&P@Al(SS) refers to the co-substituting P-doped ye’elimite with cubic system). It should be noted that the *E*_f_ of the co-substituting P-doped ye’elimite was, respectively, equaled to −0.22 and −0.18 eV for orthorhombic and cubic system (the comparison of *E*_f_ in this paper and references can be seen in Table A5), which illustrates the co-substituting models were more rational than the above single substituting models.

**Figure 13:**
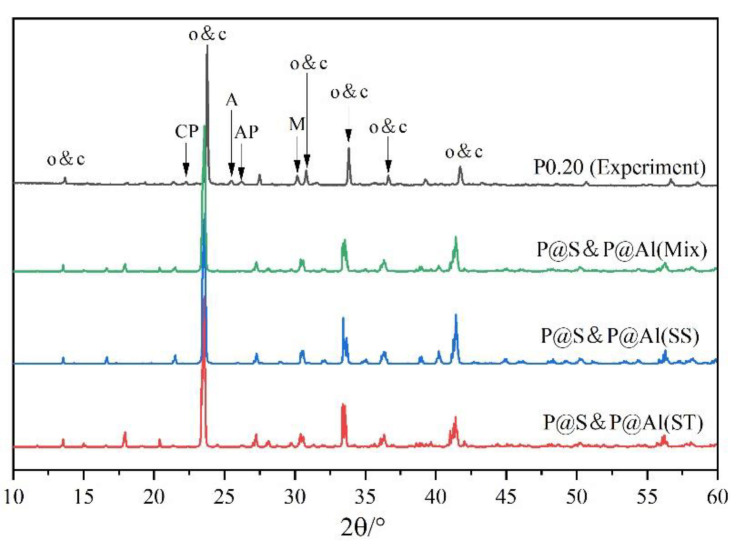
Comparison of experimental and simulated XRD patterns.

**Figure A2:**
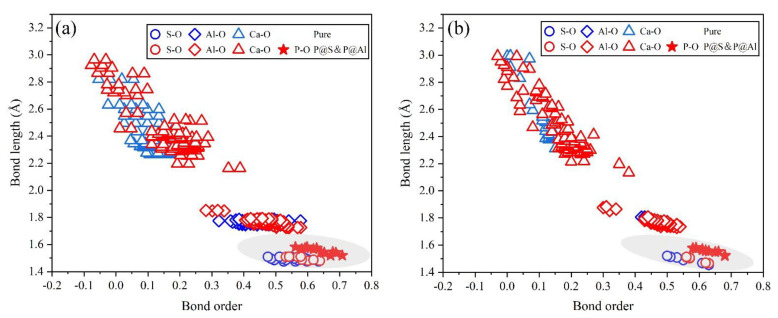
Distributions of BL–BO in pure and the most probably P-doped ye’elimite: (**a**) P@S&P@Al(ST) and (**b**) P@S&P@Al(SS).

**Figure A3:**
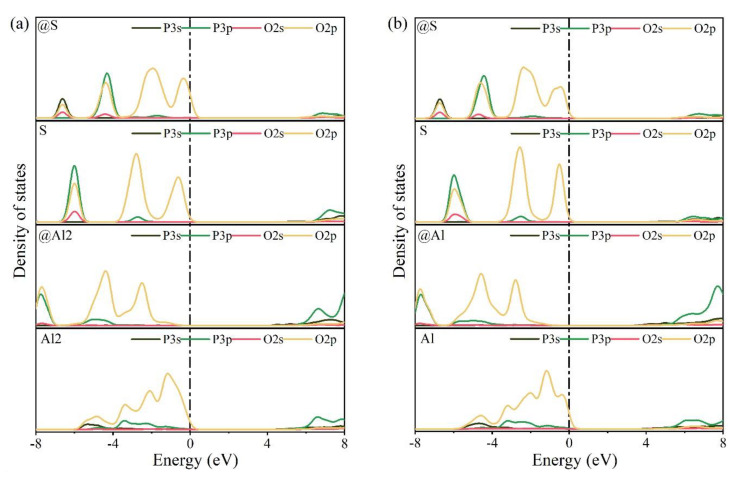
Calculation results of PDOS for pure and the most probably P-doped ye’elimite (position of the Fermi energy level (0 eV) is indicated by a dotted line), (**a**) P@S&P@Al(ST) and (**b**) P@S&P@Al(SS).

To further examine the rationality of the co-substituting P-doped ye’elimite, experimental and simulated XRD patterns were compare d in Figure 13. P@S&P@Al(Mix) refers to the mixture of P@S&P@Al(ST) and P@S&P@Al(SS), and the ratio of SS/ST was set as 0.89 which is the same as P0.20. It can be noticed that major peaks of ye’elmite for the experiment and simulation were almost coincident. Additionally, the distributions of BL–BO and PDOS were also calculated and shown in Figure A2 and Figure A3, which can also verify the rationality of the model of co-substituting P-doped ye’elimite from the aspect of electronic structure.

## 5. Conclusions

The effect of different doping amounts of P on the crystalline structures of ye’elimite was investigated, and the doping mechanism was revealed using BSEM–EDS, XRD tests, the Rietveld method, and DFT simulations. The following conclusions were obtained:

1. Experiments and Rietveld analysis confirmed that doped P entered ye’elimite to form P-doped solid solutions, resulting in increased impurity contents in clinkers, a crystal system transformation from the orthorhombic to the cubic system, and a decrease in the ye’elimite interplanar spacing.
2. Based on calculation results of *E*_f_, additional energies were required for P atoms to substitute Ca/Al atoms compared with those for substituting S atoms for both orthorhombic and cubic structures of ye’elimite. By combining the BL–BO and PDOS calculation results, the doped P atoms preferably substituted S atoms; the second possible substituted atoms were Al atoms, while there was only a slight possibility for substitution of Ca atoms.
3. The substitution of P atoms for S atoms can be verified using the elemental distribution in P-doped ye’elimite and the increasing residual CaSO_4_ contents. The crystal system transformation and a decrease in the ye’elimite interplanar spacing can also imply that the substitution of P atoms for Al atoms occurred substantially.
4. Based on analysis of phosphorus substitution preference in ye’elimite, the co-substituting P-doped ye’elimite were proposed to be the most probably models. Through values of *E*_f_, comparison of XRD patterns and electronic structural matching, the rationality of the model for co-substituting P-doped ye’elimite can be verified.

## Figures and Tables

**Table 1:** Molar ratios of raw materials and sintering conditions for different types of ye’elimite.

Nomenclature	Molar Ratios of Raw Materials	Sintering Conditions
ST	CaCO_3_: Al_2_O_3_: CaSO_4_ = 3:3:1	1300 °C for 4 h
SS	CaCO_3_: Na_2_CO_3_: Al_2_O_3_: Fe_2_O_3_: SiO_2_: CaSO_4_ = 2.8:0.1:2.8:0.1:0.2:1	1250 °C for 4 h
Px ^1^	CaCO_3_: Ca_3_(PO_4_)_2_: Al_2_O_3_:CaSO_4_ = (3–3x): x: 3:1	1300 °C for 4 h

^1^ x Corresponds to the amounts of doped phosphoric anhydride and is equal to 0.10, 0.15, and 0.20 mol in this study.

**Table 2:** COD codes and references of phases involved in Rietveld method.

Phases	Formula	COD Code	Reference
Ye’elimite (orthorhombic)	Ca_4_Al_6_SO_16_	4001772	[12]
Ye’elimite (cubic)	Ca_3.8_Na_0.2_Al_5.6_Fe_0.2_Si_0.2_SO_16_	4511960	[16]
Monocalcium aluminate	CaAl_2_O_4_	4308075	[34]
Anhydrite	CaSO_4_	5000040	[35]
Mayenite	Ca_12_Al_14_O_33_	2102955	[36]
Tricalcium aluminate	Ca_3_Al_2_O_6_	9015966	[37]
Calcium phosphate	Ca_3_(PO_4_)_2_	1517238	[38]
Calcium oxide	CaO	7200686	[39]
Aluminum phosphate	AlPO_4_	9006404	[40]

**Table 3:** RQPA results (wt.%) for clinkers containing P-doped ye’elimite.

Phases	Samples			
	ST	P0.10	P0.15	P0.20
Orthorhombic ye’elimite	96.4	75.8	61.5	44.7
Cubic ye’elimite	-	18.5	30.2	39.8
Calcium aluminate phases ^1^	2.8	5.7	6.4	11.6
Anhydrite	-	-	0.6	1.2
Calcium oxide	0.8	-	-	-
Calcium/aluminum phosphate ^2^	-	-	1.3	2.7
Ratio of cubic ye’elimite to orthorhombic ye’elimite	0	0.24	0.49	0.89
Total contents of ye’elimite	96.4	94.3	91.7	84.6

^1^ Calcium aluminate phases included Ca_12_Al_14_O_33_, CaAl_2_O_4_, CaAl_4_O_7_, and Ca_3_Al_2_O_4_. ^2^ Calcium/aluminum phosphate referred to Ca_3_(PO_4_)_2_/AlPO_4_, respectively.

**Table 4:** Radii of ions involved in P-doped ye’elimite [55].

Ions	Coordination Numbers	Radius (pm)
P^5+^	4	17
	5	29
	6	38
Ca^2+^	6	100
	7	106
	8	112
S^6+^	4	12
Al^3+^	4	39

## Data Availability

Not applicable.

## Appendix A

**Table A1:** K-point grids for simulation models.

Phases	Supercells	Relax	PDOS
Orthorhombic ye’elimite	1 × 1 × 1	2 × 2 × 3	4 × 4 × 5
Cubic ye’elimite	2 × 1 × 1	1 × 3 × 3	3 × 5 × 5

**Table A2:** Details on Gibbs free energies involved in calculations for *μ*_P_ and *μ*_0_.

Oxides	P_2_O_5_	Al_2_O_3_	CaO	SO_3_	O_2_
Identification of Materials Project	mp-2452	mp-1143	mp-2605	mp-561397	mp-1009490
Space group	FDD2	R-3C	Fm-3m	P21/c	C2/M
Cells	1 × 1 × 1	1 × 1 × 1	1 × 1 × 1	1 × 1 × 1	1 × 1 × 1
Number of atoms	2	2	1	4	1
a/Å	16.59	4.81	4.84	13.43	4.72
b/Å	8.17	4.81	4.84	4.18	4.73
c/Å	5.55	13.12	4.84	9.75	4.96
α/°	90.00	90.00	90.00	90.00	90.00
β/°	90.00	90.00	90.00	153.05	123.11
γ/°	90.00	120.00	90.00	90.00	90.00
V/Å3	751.81	262.26	113.33	247.98	93.14
*G*_Bulk_ (eV)	−98.40	−74.83	−12.81	−92.84	−9.08
*G* (eV)	−49.20	−37.42	−12.81	−23.21	−9.08

**Table A3:** Relaxed lattice parameters of the stable configurations including pure and P-doped clinker phases.

Configurations		Lattice Parameters						
		a/Å	b/Å	c/Å	α/°	β/°	γ/°	volume/Å^3^
P@ST (1 × 1 × 1)	Ref. [12]	13.04	13.04	9.17	90.00	90.00	90.00	1557.78
	Pure ^1^	13.09	13.26	9.25	90.00	90.00	90.00	1606.36
	@Al1	12.97	13.04	9.52	90.00	90.00	90.06	1609.79
	@Al2	13.17	13.33	9.02	90.04	90.00	90.00	1584.44
	@Ca	13.04	13.22	9.33	89.55	89.40	89.50	1606.85
	@S	13.04	13.22	9.32	89.59	89.45	89.57	1607.25
P@SS (2 × 1 × 1)	Ref [16]	18.42	9.21	9.21	90.00	90.00	90.00	779.96
	Pure ^1^	18.55	9.27	9.23	90.02	89.98	89.37	1586.91
	@Al	19.04	9.30	9.04	91.34	92.01	86.71	1595.49
	@Ca	18.75	9.30	9.14	89.34	90.35	89.34	1593.46
	@S	18.53	9.26	9.23	89.87	90.13	89.49	1584.47

^1^ Lattice parameters of pure orthorhombic and cubic ye’elimite used in this paper were obtained through optimizing the crystal structures in Ref. [12] and Ref. [16], respectively.

**Table A4:** Total energy (*E*_t_), Bonding energy (*E*_b_) and defect formation energy (*E*_f_) of substituted elements X (X = Ca, Al, and S).

Sample	Configurations	*E*_t_/eV	*E*_b_ ^1^/eV	*E*_f_/eV
P@ST	Pure	−48,268.83	−770.23	-
	@Al1	−48,477.11	−764.33	4.30
	@Al2	−48,477.13	−764.35	4.29
	@Ca	−46,685.89	−763.60	8.30
	@S	−48,103.16	−775.02	1.27
P@SS	Pure	−48,268.60	−770.00	-
	@Al	−48,477.61	−764.83	3.94
	@Ca	−46,684.65	−762.36	8.80
	@S	−48,103.06	−774.92	1.20

^1^ The formula for calculating the binding energy is: *E*_b_ = *E*_t_ − *N*_Al_*E*_Al_ − *N*_O_*E*_O_ − *N*_Ca_*E*_Ca_ − *N*_S_*E*_S_ − *N*_P_*E*_P_. where *E*_b_ is the bonding energy, *E*_t_ is the total energy of unit cell, and *E*_Al_, *E*_O_, *E*_Ca_, *E*_S_ and *E*_P_ are the energies of pseudo atomic Al, O, Ca, S and P respectively. *N*_Al_, *N*_O_, *N*_Ca_, *N*_S_ and *N*_P_ are the numbers of Al, O, Ca, S and P atoms in the unit cell, respectively.

**Table A5:** Comparison of *E*_f_ in this paper and references.

Crystal System	Configurations	Value of *E*_f_/eV	Sources
Orthorhombic system	P@S&P@Al	−0.22	This paper
Cubic system	P@S&P@Al	−0.18	This paper
Cubic system	Ba@Ca	−1.34	Ref. [26]
Cubic system	Pb@Ca	−0.18	Ref. [27]
